# Complete eversion and prolapse of bladder following pulling out of a Foley catheter concurrent with uterine prolapse

**DOI:** 10.4103/0970-1591.36727

**Published:** 2007

**Authors:** Amulya M. Acharya, D. R. Mishra

**Affiliations:** Department of Surgery, Ispat General Hospital, Rourkela, Orissa - 769 005, India; * Department of Obstetrics and Gynecology, Ispat General Hospital, Rourkela, Orissa - 769 005, India

**Keywords:** Bladder, eversion, prolapse

## Abstract

Complete eversion and transurethral prolapse of the urinary bladder is rare. We report a case of complete eversion and prolapse of bladder that occurred due to self pulling out of an indwelling Foley catheter in a 72-year-old woman. She presented with retention of urine concurrent with complete uterine procidentia. An indwelling Foley catheter was given to relieve the retention. The senile lady pulled out the catheter resulting in complete transurethral prolapse with bladder eversion. Under injection Midazolam sedation and with application of xylocain jelly the prolapsed bladder could be reduced manually back through the urethra. Definite corrective surgery was done later for the uterine prolapse.

## INTRODUCTION

Complete eversion and transurethral prolapse of urinary bladder is rare. Few cases have been reported this occurring in elderly woman, concurrent with uterovaginal prolapse, following labor and eversion through vesicovaginal fistulas. We report a rare case of complete eversion and prolapse of bladder through the urethra following pulling out of an indwelling Foley catheter given to an elderly women admitted for retention of urine concurrent with uterovaginal prolapse.

## CASE REPORT

**Figure 1:**
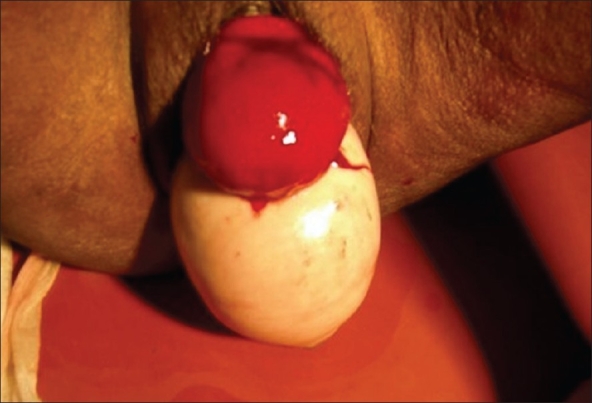
Red edematous, slimy, pyriform mass of everted and prolapsed bladder sitting over the uterine procidentia

**Figure 2:**
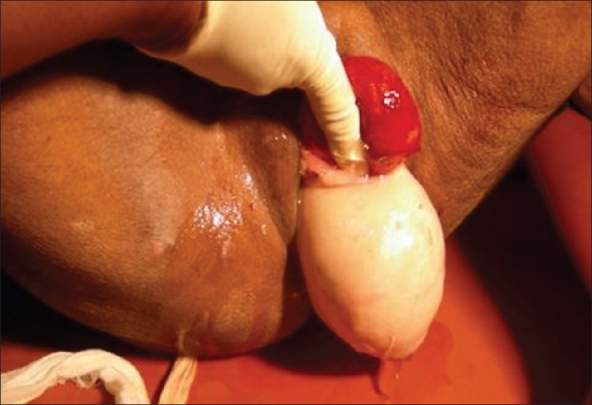
Finger inside posteriorly placed urethral orifice

A 72-year-old woman was admitted for retention of urine. She was a multiparous elderly lady and examination revealed longstanding complete uterine prolapse. An indwelling Foley catheter was given to relieve the retention. Next day, the senile lady pulled out the catheter herself resulting in complete eversion and prolapse of the bladder. Physical examination revealed a red edematous, slimy, pyriform mass 6 cm in diameter sitting over the completely prolapsed uterus [Figure 1]. The mass was separate from the uterine mass and had an orifice posteriorly placed [Figure 2]. On introducing a catheter through the orifice about 10 ml urine was obtained. A diagnosis of complete eversion and prolapse of bladder was made.

**Figure 3:**
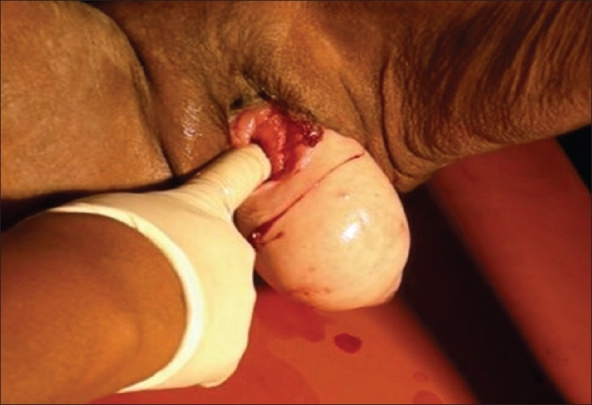
Prolapsed bladder being completely reduced back through the urethra

Patient was sedated with injection Midazolam and after applying a liberal amount of xylocain jelly, the everted bladder was reduced back through the urethra [Figure 3]. External urethral meatus showed a small tear, she was catheterized again. A definitive corrective surgery for the uterine prolapse was carried out later.

## DISCUSSION

Complete transurethral eversion and prolapse of bladder is rare and the pathogenesis is not much known. Some earlier reports mention complete transurethral inversion of bladder occurring during labor.[1] Few reported as complication of vesicovaginal fistula.[2,3] Senility, obesity, multiple labor could be possible causes of complete eversion of bladder.[4] Uterovaginal prolapse with laxity of pelvic floor are seen associated with this condition.[5] Delayed management may lead to serious conditions like acute renal failure.[3] In one case concurrent adenocarcinma in the thickened wall of the everted urinary bladder could be demonstrated by MRI.[6]

In our case, traumatic pulling out of the Foley catheter precipitated the complete eversion and prolapse of the urinary bladder in the previous weak and lax pelvic floor concurrent with the existing uterine procidentia.
